# SWI/SNF proteins as targets in cancer therapy

**DOI:** 10.1186/s13045-014-0081-5

**Published:** 2014-11-13

**Authors:** Santiago Schiaffino-Ortega, Carlos Balinas, Marta Cuadros, Pedro P Medina

**Affiliations:** Department of Biochemistry and Molecular Biology, University of Granada, Granada, Spain; GENyO, Centre for Genomics and Oncological Research: Pfizer / University of Granada / Andalusian Regional Government, Granada, Spain

**Keywords:** SWI/SNF complex, Cancer therapy, Epigenetic, Tumor suppressor, Synthetic lethality, Leukemia, Lung cancer, BRG1, SMARCA4, BRM, SMARCA2, MAX, MYC

## Abstract

Recent identification of synthetic lethal interactions involving several proteins of the SWI/SNF complex discussed in this Research Highlight has opened the possibility of new cancer therapeutic approaches.

Research over the two last decades has shown that epigenetic deregulation is a common feature in carcinogenesis. With the latest advances in cancer genome sequencing, the occurrence of inactivating mutations in SWI/SNF chromatin-remodeling genes in several tumor types has attracted a great deal of interest. The SWI/SNF complex alters the interactions between DNA and histones using the energy of ATP hydrolysis, modifying the availability of DNA’s information to the cellular machinery. In this way, the SWI/SNF complex can modify gene expression, such as by controlling the accessibility of DNA to transcription factors, thereby controlling transcription as a whole.

Various studies have determined that genes encoding subunits of the SWI/SNF chromatin-remodeling complex are mutated in cancer about 20% of the time. Initial insights into the role of the SWI/SNF complex in tumorigenesis arose from the identification of the core subunit SMARCB1/SNF5 in malignant rhabdoid tumors, following demonstration of its tumor suppressor function in cell lines and animal models. Subsequently, mutations and/or loss of expression of the catalytic subunit SMARCA4 have been reported predominantly in non-small cell lung cancer (NSCLC), as well as other cancers. Furthermore, mutations in the accessory core subunit ARID1A have been reported in ovarian clear cell and endometrial carcinomas, among other cancers, and PBRM mutations have been reported in clear renal cell carcinomas.

## Synthetic lethality strategies dependent on the mutational status of SWI/SNF complex proteins

Recent understanding of the role and importance of the SWI/SNF complex in tumor development has opened the door to new potential therapeutic strategies based on the concept of synthetic lethality [1].

Synthetic lethality is defined as a type of genetic interaction where the co-occurrence of two genetic events results in organismal or cellular death. The term also can be applied to cancer biology. During their transformation, cancer cells acquire genetic and epigenetic variations that distinguish them from their wild-type counterparts. Consequently, cancer cells have been reprogrammed, exposing new genetic and epigenetic vulnerabilities. Synthetic lethal interaction partners of cancer-associated molecular changes should therefore offer therapeutic opportunities. In recent years, synthetic lethality has attracted attention in the field of oncology, as it provides a new angle for therapy and may explain the sensitivity of cancer cells to certain drugs.

**Figure 1:**
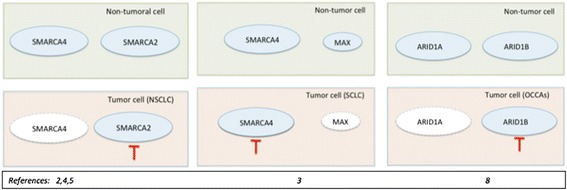
**Recently proposed therapeutic models that exploit synthetic lethality strategies involving SWI**/**SNF proteins.** The red inhibition line indicates the therapeutic strategy in the specific tumor cells assayed in the reports referenced in the figure. Proteins with dotted lines indicate expression inactivation. NSCLC: non-small cell lung cancer, SCLC: small cell lung cancer, OCCAs: ovarian clear cell adenocarcinomas.

In this Research Highlight, we will discuss some of the potential therapeutic strategies that can be developed by targeting the SWI/SNF complex using the concept of synthetic lethality [2-5] (Figure 1).

## Synthetic lethality in tumors with SMARCA4 mutations after inhibition of SMARCA2

The SWI/SNF complex contains one of the two mutually exclusive DNA-dependent ATPases, SMARCA4 (also known as BRG1), and SMARCA2 (also known as BRM). Although SMARCA4 and SMARCA2 display high homology and presumably have overlapping functions, other observations suggest that they have different roles in cancer. For instance, tumor sequencing has unveiled some of these functional differences: SMARCA2 inactivation by mutation is infrequent in tumor development; however, SMARCA4 has been found mutated in primary tumors and cancer cell lines. The first somatic mutation of SMARCA4 was described in an NSCLC tumor [6], and expression inactivation by mutation was also frequently found in NSCLC cell lines [7]. Using these findings, three research groups sought to test whether depletion of SMARCA2 might be synthetically lethal in *SMARCA4*-mutant lung tumors [2,4,5].

In a pioneer study, Oike *et al.* screened siRNA against *SMARCA2* in a panel of *SMARCA4* mutant NSCLC cell lines and found that the inhibition of S*MARCA2* significantly decreased cell viability compared with control siRNA, whereas *SMARCA4* wild-type cells were unaffected. They extended these results to a xenograft mouse model of NSCLC [5]. Independently, another group studied the same relationship and found that *SMARCA4* inactivation leads to greater incorporation of the nonessential SMARCA2 subunit into the SWI/SNF complex [2]. Wilson and colleagues observed that this residual SWI/SNF complex exists in SMARCA4-mutant cell lines and plays essential roles in cellular proliferation. Additionally, using data from loss-of-function screening of 165 cancer cell lines, the authors identified SMARCA2 as the top essential gene, even more than *TP53*, in SMARCA4 mutant cancer cell lines [2].

Hoffman *et al.* also identified SMARCA2 as being essential for the growth of tumor cells that harbor loss of function mutations in SMARCA4 [4]. The researchers depleted SMARCA2 in SMARCA4-deficient cancer cell lines and xenograft models and observed cycle arrest, induction of senescence, and increased level of the repressive marker H3K9me3. The authors proposed that such synthetic lethality might be explained by paralog insufficiency, in which loss of one family member unveils critical dependence on paralogous subunits [4].

## Synthetic lethality in tumors with MAX mutations after inhibition of SMARCA4

Recently, Romero and colleagues found another putative synthetic lethality that also involves *SMARCA4* and that could be exploited as a putative therapy in small cell lung cancer (SCLC) [3]. Studying the inactivation of MYC-associated factor X (*MAX*) gene in SCLC, they found that about 6% of the tumors have homozygous and tumor-specific mutations in *MAX.* Interestingly, the authors observed that the alterations in *MAX* and amplification of the MYC genes were mutually exclusive, and none of the *MAX*-mutant cells carried concomitant mutations of *SMARCA4*. The researchers observed that *SMARCA4* was able to regulate *MAX* expression by binding specifically to its promoter, and that depletion of *SMARCA4* strongly hinders cell growth, specifically in *MAX*-deficient cells, indicating a synthetic lethal interaction that could be therapeutically exploited [3].

## Synthetic lethality in tumors with ARID1A mutations after inhibition of ARID1B

Recent studies have revealed that another member of the SWI/SNF complex, *ARID1A*, which encodes AT-rich interactive domain 1A, is frequently mutated across a variety of human cancers (including ovarian, breast, and lung, among others) and also has *bona fide* tumor suppressor properties. Using a broad screening approach, Helming *et al.* identified *ARID1B*, an *ARID1A* homolog whose gene product is mutually exclusive with *ARID1A* in SWI/SNF complexes [8], as the preferentially required gene for the survival of *ARID1A*-mutant ovarian cancer cell lines. The authors showed that loss of *ARID1B* in *ARID1A*-deficient backgrounds destabilizes SWI/SNF and impairs proliferation in both cancer cells and primary cells, thus acting as a synthetic lethal and opening new therapeutic opportunities.

## Small-molecule drugs to therapeutically exploit the SWI/SNF complex

Epigenetic proteins have been intently pursued as targets for cancer therapy. However, it was thought that SWI/SNF complex proteins were not good targets for cancer therapy due to the tumor suppressor activities of the complex. Nevertheless, the research highlighted here describing synthetic lethality interactions between members of the SWI/SNF complex with putative therapeutic applications may have changed this point of view.

A new therapeutic approach includes the use of inhibitors that specifically target bromodomains. The proof of concept of these small cell-permeable inhibitors came from a report where the inhibitor I-BET151 was able to bind specifically to the bromodomains of BRD3/4 and be therapeutically active in MLL-fusion leukemia [9]. The bromodomain is a highly conserved motif of 110 amino acids that is bundled into four anti-parallel α-helices that are able to bind to acetylated lysines in the histones of nucleosomal chromatin. These domains are therefore able to read information of the histone code. Importantly, the SWI/SNF complex has several proteins with these druggable bromodomains, including SMARCA4, SMARCA2, BRD9, and PBRM1. In this way, for example, the specific inhibition of SMARCA2 could have a therapeutic benefit in *SMARCA4* mutant tumors. In the same way, the inhibition of SMARCA4 could have therapeutic benefits in *MAX* mutant tumors.

Additionally, these drugs may be exploited in other genetic contexts where the SWI/SNF activities are necessary to maintain the tumor phenotype. For example, recent reports have observed that while in most of the tumor types studied, *SMARCA4* is known to have a tumor suppressive function, leukemia cells instead rely on *SMARCA4* to support their oncogenic transcriptional program [10,11].

## Conclusions

The recent identification of synthetic lethal interactions involving several subunits of the SWI/SNF complex has opened the possibility of new therapeutic approaches that can be exploited using a new generation of epigenetic inhibitors. A recent report suggested that the incipient microRNA-based technology could be also in the arsenal to target SWI/SNF proteins [12]. As the field of molecular therapeutics on epigenetic proteins rapidly expands, several features of protein function will need to be considered, including possible off-target inhibition or uncontrolled transcriptional derepression of genes, altered hematopoiesis, and immunosuppression or reactivation of latent viruses. Despite these possible pitfalls, the harnessing of SWI/SNF for clinical applications holds great promise, and oncologists are increasingly interested in this therapeutic potential.

## References

[1] Hartwell LH, Szankasi P, Roberts CJ, Murray AW, Friend SH (1997). Integrating genetic approaches into the discovery of anticancer drugs. Science.

[2] Wilson BG, Helming KC, Wang X, Kim Y, Vazquez F, Jagani Z, Hahn WC, Roberts CW (2014). Residual complexes containing SMARCA2 (BRM) underlie the oncogenic drive of SMARCA4 (BRG1) mutation. Mol Cell Biol.

[3] Romero OA, Torres-Diz M, Pros E, Savola S, Gomez A, Moran S, Saez C, Iwakawa R, Villanueva A, Montuenga LM, Kohno T, Yokota J, Sanchez-Cespedes M (2014). MAX inactivation in small cell lung cancer disrupts MYC-SWI/SNF programs and is synthetic lethal with BRG1. Cancer Discov.

[4] Hoffman GR, Rahal R, Buxton F, Xiang K, McAllister G, Frias E, Bagdasarian L, Huber J, Lindeman A, Chen D, Romero R, Ramadan N, Phadke T, Haas K, Jaskelioff M, Wilson BG, Meyer MJ, Saenz-Vash V, Zhai H, Myer VE, Porter JA, Keen N, McLaughlin ME, Mickanin C, Roberts CW, Stegmeier F, Jagani Z (2014). Functional epigenetics approach identifies BRM/SMARCA2 as a critical synthetic lethal target in BRG1-deficient cancers. Proc Natl Acad Sci U S A.

[5] Oike T, Ogiwara H, Tominaga Y, Ito K, Ando O, Tsuta K, Mizukami T, Shimada Y, Isomura H, Komachi M, Furuta K, Watanabe S, Nakano T, Yokota J, Kohno T (2013). A synthetic lethality-based strategy to treat cancers harboring a genetic deficiency in the chromatin remodeling factor BRG1. Cancer Res.

[6] Medina PP, Carretero J, Fraga MF, Esteller M, Sidransky D, Sanchez-Cespedes M (2004). Genetic and epigenetic screening for gene alterations of the chromatin-remodeling factor, SMARCA4/BRG1, in lung tumors. Genes Chromosomes Cancer.

[7] Medina PP, Romero OA, Kohno T, Montuenga LM, Pio R, Yokota J, Sanchez-Cespedes M (2008). Frequent BRG1/SMARCA4-inactivating mutations in human lung cancer cell lines. Hum Mutat.

[8] Helming KC, Wang X, Wilson BG, Vazquez F, Haswell JR, Manchester HE, Kim Y, Kryukov GV, Ghandi M, Aguirre AJ, Jagani Z, Wang Z, Garraway LA, Hahn WC, Roberts CW (2014). ARID1B is a specific vulnerability in ARID1A-mutant cancers. Nat Med.

[9] Dawson MA, Prinjha RK, Dittmann A, Giotopoulos G, Bantscheff M, Chan WI, Robson SC, Chung CW, Hopf C, Savitski MM, Huthmacher C, Gudgin E, Lugo D, Beinke S, Chapman TD, Roberts EJ, Soden PE, Auger KR, Mirguet O, Doehner K, Delwel R, Burnett AK, Jeffrey P, Drewes G, Lee K, Huntly BJ, Kouzarides T (2011). Inhibition of BET recruitment to chromatin as an effective treatment for MLL-fusion leukaemia. Nature.

[10] Shi J, Whyte WA, Zepeda-Mendoza CJ, Milazzo JP, Shen C, Roe JS, Minder JL, Mercan F, Wang E, Eckersley-Maslin MA, Campbell AE, Kawaoka S, Shareef S, Zhu Z, Kendall J, Muhar M, Haslinger C, Yu M, Roeder RG, Wigler MH, Blobel GA, Zuber J, Spector DL, Young RA, Vakoc CR (2013). Role of SWI/SNF in acute leukemia maintenance and enhancer-mediated Myc regulation. Genes Dev.

[11] Buscarlet M, Krasteva V, Ho L, Simon C, Hebert J, Wilhelm B, Crabtree GR, Sauvageau G, Thibault P, Lessard JA (2014). Essential role of BRG, the ATPase subunit of BAF chromatin remodeling complexes, in leukemia maintenance. Blood.

[12] Coira IF, Rufino-Palomares EE, Romero OA, Peinado P, Metheetrairut C, Boyero-Corral L, Carretero J, Farez-Vidal E, Cuadros M., Reyes-Zurita F, Lupiáñez JA, Sánchez-Cespedes M., Slack FJ Medina PP: **Expression inactivation of SMARCA4 by microRNAs in lung tumors.** *Human Molecular Genetics* 2014, doi:10.1093/hmg/ddu554 10.1093/hmg/ddu554 PMC4321447 25355421

